# Rac-Mediated Macropinocytosis of Extracellular Protein Promotes Glucose Independence in Non-Small Cell Lung Cancer

**DOI:** 10.3390/cancers11010037

**Published:** 2019-01-02

**Authors:** Cindy Hodakoski, Benjamin D. Hopkins, Guoan Zhang, Taojunfeng Su, Zhe Cheng, Roxanne Morris, Kyu Y. Rhee, Marcus D. Goncalves, Lewis C. Cantley

**Affiliations:** 1 Meyer Cancer Center, Weill Cornell Medicine-New York Presbyterian Hospital, New York, NY 10021, USA; cih2002@med.cornell.edu (C.H.); beh2020@med.cornell.edu (B.D.H.); mdg9010@med.cornell.edu (M.D.G.); 2 Proteomics and Metabolomics Core Facility, Weill Cornell Medicine, New York, NY 10021, USA; guz2004@med.cornell.edu (G.Z.); tas2033@med.cornell.edu (T.S.); zhe.cheng@nyu.edu (Z.C.); 3 Division of Infectious Diseases, Weill Department of Medicine, Weill Cornell Medicine, 1300 York Ave A-421, New York, NY 10065, USA; roxanne.morris22@gmail.com (R.M.); kyr9001@med.cornell.edu (K.Y.R.); 4 Division of Endocrinology, Department of Medicine, Weill Cornell Medicine, New York, NY 10065, USA

**Keywords:** macropinocytosis, Rac, metabolism, glucose

## Abstract

Cancer cells can adapt to nutrient poor conditions by rewiring their metabolism and using alternate fuel sources. Identifying these adaptive metabolic pathways may provide novel targets for cancer therapy. Here, we identify a subset of non-small cell lung cancer (NSCLC) cell lines that survive in the absence of glucose by internalizing and metabolizing extracellular protein via macropinocytosis. Macropinocytosis is increased in these glucose independent cells, and is regulated by phosphoinositide 3-kinase (PI3K) activation of Rac-Pak signaling. Furthermore, inhibition of Rac-dependent macropinocytosis blocks glucose-independent proliferation. We find that degradation of internalized protein produces amino acids, including alanine, which generates TCA cycle and glycolytic intermediates in the absence of glucose. In this process, the conversion of alanine to pyruvate by alanine transaminase 2 (ALT2) is critical for survival during glucose starvation. Collectively, Rac driven macropinocytosis of extracellular protein is an adaptive metabolic pathway used by a subset of lung cancers to survive states of glucose deprivation, and may serve as a potential drug target for cancer therapy.

## 1. Introduction

It has been well established that tumor cells have altered metabolism compared to non-proliferating cells. Many cancer cells import large amounts of glucose and convert it to lactate, despite the presence of oxygen, in a phenomenon known as the “Warburg effect” or aerobic glycolysis [1,2]. Glycolysis produces intermediates that fuel biosynthetic pathways to produce amino acids, nucleotides, and lipids [3]. However, recent reports have demonstrated that glucose levels vary in solid tumors, and glucose concentrations are often decreased in tumors compared to the surrounding normal tissue [4,5,6,7]. This deficiency of glucose in the tumor microenvironment makes it necessary for cancer cells to adopt alternative metabolic pathways to support tumor growth.

In the absence of glucose, tumors rely on alternate fuel sources to support cell growth in nutrient-poor environments [8,9]. Some cancer cells are dependent on glutaminolysis to generate glutamate and α-ketoglutarate which can drive the tricarboxylic acid (TCA) cycle and ATP production. This pathway also provides both nitrogen and carbon for macromolecule biosynthesis [10,11,12,13]. In addition, numerous reports have shown that cancer cells express gluconeogenic enzymes that enable them to utilize non-glucose carbons to fuel biosynthetic pathways when glucose is unavailable. It has been reported that the gluconeogenic enzyme phosphoenolpyruvate carboxykinase (PEPCK) is critical for the growth of several cancers, including lung and colon cancers. [14,15,16,17]. Also, the gluconeogenic enzyme fructose-1,6-bisphosphatase is upregulated to support glucose-independent proliferation of brain-metastatic breast cancer cells, while conversely being downregulated in glycolytic renal cell carcinomas [18,19].

Lung cancer is the leading cause of cancer-related deaths worldwide. Non-small cell lung cancer (NSCLC) accounts for approximately 85% of lung cancers [20]. Common mutational events in NSCLC, including *KRAS* mutation and *LKB1* loss, result in altered metabolic demands. For example, the growth of *Kras^G12D^;p53^−/−^* murine lung tumors requires the uptake of branched-chain amino acids [21]. Furthermore, loss of LKB1 in lung cancer cells leads to increased uptake of both glucose and glutamine as well as increased flux through glycolysis and the TCA cycle [22]. The plasticity of lung cancer metabolism allows these cells to survive in the absence of glucose by relying on alternative metabolic pathways. Uncovering these metabolic pathways may help identify potential targets for therapeutic intervention. Therefore, we set out to identify novel metabolic dependencies in NSCLC cells during glucose withdrawal.

## 2. Results

### 2.1. Glucose-Independent NSCLC Cells Require Extracellular Protein for Growth During Glucose Withdrawal

**Figure 1:**
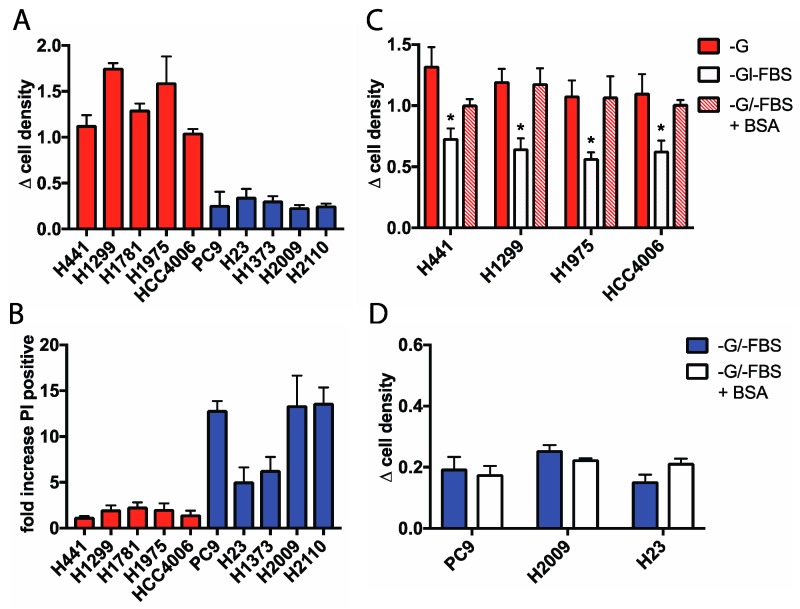
(**A**) Glucose independent (red) and glucose addicted (blue) NSCLC cell lines were cultured in glucose-free media (GFM) for 48 h. For all experiments, “change in cell density” is calculated by measuring the change in crystal violet staining from 0 to 48 h. Error bars indicate ± SEM of at least three experiments. (**B**) Cell death of NSCLC cells cultured in GFM for 24 h as measured by propidium iodide (PI) uptake. Values shown are the fold increase in PI positive cells in glucose-free media compared to cells cultured in full-glucose medium. Error bars indicate ± SEM of three independent experiments. (**C**) Change in cell density of glucose independent cells cultured for 48 h in GFM with or without dialyzed FBS (dFBS) or 2% albumin (BSA), “G”, glucose. Error bars indicate ± SEM of three independent experiments. Significance was calculated using ANOVA with Holm-Sidak multiple comparisons to –G condition, * *p* < 0.05. (**D**) Change in cell density of glucose addicted cells cultured in GFM alone or supplemented with 2% albumin for 48 h. Error bars indicate ± SEM of at least two independent experiments.

To identify how lung cancer cells adapt their metabolism to overcome glucose starvation, we cultured a panel of NSCLC cells lines in glucose-free medium containing dialyzed fetal bovine serum (dFBS), and measured cell viability following 48 h of glucose deprivation. A subset of “glucose independent” cell lines, including H1299, H441, H1975, H1781, and HCC4006 continued to proliferate in the absence of glucose, while “glucose addicted” PC9, H23, H1373, H2009, and H2110 cells demonstrated significant decreases in cell viability and underwent significant cell death as determined by propidium iodide staining (Figure 1A,B). Interestingly, glucose independent cells were dependent on the presence of serum in the media to sustain glucose free proliferation, as removal of dFBS resulted in a significant decrease in cell viability upon glucose withdrawal (Figure 1C). This result suggests that these cells are reliant on a component in serum as either a growth factor and/or a metabolic fuel for growth. A major component of blood serum is soluble protein, with albumin being the most abundant. Furthermore, albumin is found at high concentrations in tissue and tumor samples [23,24]. To determine if cells required extracellular protein to grow when glucose starved, we supplemented glucose free medium with 2% fatty acid-free bovine serum albumin to mimic the physiological concentrations of albumin in serum. Indeed, the addition of albumin rescued cell viability in the absence of glucose and serum (Figure 1C), suggesting that lung cancer cells may internalize extracellular protein and use it as a metabolic fuel when glucose is unavailable. Conversely, the addition of albumin did not increase the viability of the cell lines that are addicted to glucose (Figure 1D), suggesting that only glucose independent cells can utilize extracellular protein as a fuel source.

### 2.2. Macropinocytosis Is Increased in Glucose Independent Cells and Is Required for Growth in the Absence of Glucose

**Figure 2:**
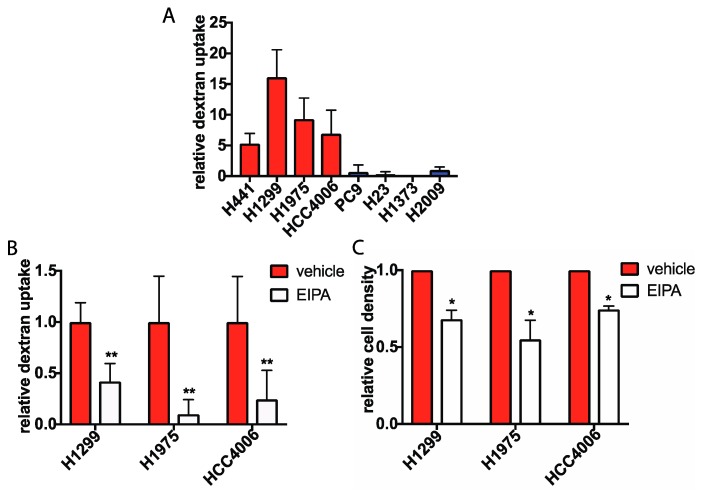
Macropinocytosis is increased in glucose independent cells and is required for growth in the absence of glucose. (**A**) Cells were cultured in GFM for 3 h prior to the addition of 1 mg/mL FITC-dextran for 1 h. Levels of FITC-dextran uptake in glucose independent (blue) and glucose addicted (red) cells was quantified and normalized to the cell line H2009. (**B**) Quantification of the levels of FITC-dextran uptake in glucose starved glucose independent cells treated with either vehicle or 10 μm EIPA for 1 h prior to incubation with FITC-dextran. Data is normalized to vehicle treated cells. For (**A**,**B**), error bars indicate ± SD of 10 fields scored containing at least 10 cells. (**C**) Relative density of glucose starved cells treated with vehicle or 10 μm EIPA for 48 h as determined by crystal violet staining. Data is normalized to density of vehicle treated cells at 48 h; error bars indicate ± SEM of three independent experiments. Significance was calculated using Student’s *t*-test, * *p* < 0.05, ** *p* < 0.005.

One mechanism by which cells can internalize large extracellular components, including proteins, is via macropinocytosis. In this endocytic process, extracellular fluid and its soluble components are internalized through the formation of actin dependent membrane protrusions, which form vesicles known as macropinosomes that can fuse with lysosomes [25]. Recent reports have shown that macropinocytosis of extracellular proteins provides amino acids as a fuel source for Ras transformed cancer cells, and is also a critical source of amino acids for nutrient poor pancreatic cancer cells in vivo [5,26,27]. Therefore, we hypothesized that glucose independent cells employ macropinocytosis to internalize extracellular proteins, while glucose addicted cells do not. To determine if the levels of macropinocytosis during glucose starvation differed between glucose independent and glucose addicted cells, we incubated glucose starved cells with fluorescein isothiocyanate (FITC) labeled, 70 kDa high molecular weight dextran, a well-established marker of macropinocytosis [28]. The internalization of extracellular FITC-dextran in vesicles was then quantified by fluorescence microscopy to determine the macropinocytotic activity in the cell. Indeed, the uptake of FITC-dextran in glucose independent cells was significantly higher compared to glucose addicted cells in the absence of glucose (Figure 2A, Figure S1).

We next treated cells with a specific inhibitor of macropinocytosis, ethylisopropylamiloride (EIPA) [29] to determine if glucose-free cell proliferation was dependent on protein internalization via macropinocytosis. Treatment of glucose independent cells with EIPA resulted in decreased FITC-dextran uptake, confirming that protein is being internalized via this process. (Figure 2B, Figure S2). Furthermore, EIPA treatment lead to a significant decrease in cell viability during glucose starvation (Figure 2C). This suggests that macropinocytosis of protein is critical for cell survival during glucose withdrawal.

### 2.3. Rac Signaling is Elevated in Glucose Independent Cells and Inhibition of Rac Decreases Macropinocytosis and Glucose-Free Survival

**Figure 3:**
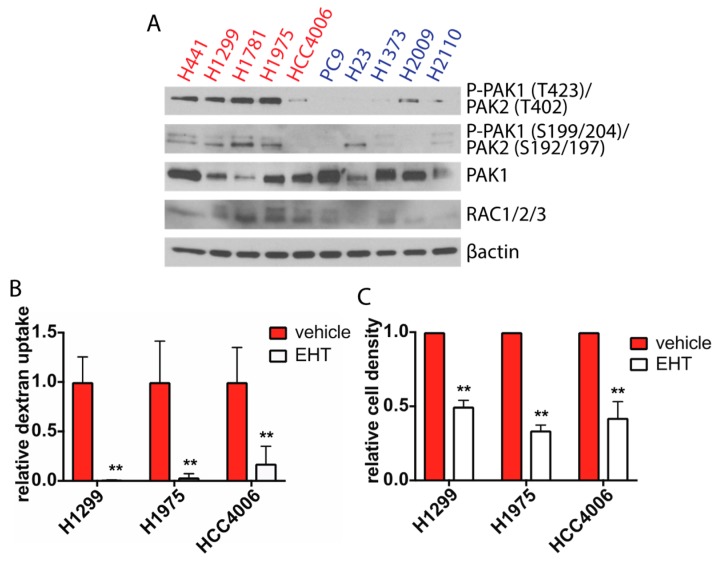
Rac signaling is increased in glucose independent cells and Rac inhibition decreases macropinocytosis and glucose-free growth. (**A**) Immunoblot analysis of Rac signaling activity via Pak autophosphorylation at Ser 199/204 (Pak1)/Ser192/197 (Pak2) and Thr-423 (Pak1)/Thr402 (Pak2). Glucose independent (red) and glucose addicted (blue) cells were cultured in GFM for 6 h before lysate collection. (**B**) Quantification of the levels of FITC-dextran uptake in glucose starved glucose independent cells treated with either vehicle or 5 μm EHT 1864 (EHT) 1 h prior to incubation with FITC-dextran. Data is normalized to vehicle treated cells, error bars indicate ± SD of 10 fields scored containing at least 10 cells. (**C**) Relative density of glucose starved cells treated with vehicle or 5 μm EHT 1864 for 48 h as determined by crystal violet staining. Data is normalized to density of vehicle treated cells at 48 h; error bars indicate ± SEM of three independent experiments. Significance was calculated using Student’s *t*-test, ** *p* < 0.005.

It has been previously reported that Rac signaling is an important regulator of macropinocytosis in macrophages, dendritic cells, and fibroblasts [30,31,32,33]. One of the critical downstream targets of Rac is the serine/threonine kinase p21-activated kinase (Pak), which promotes actin cytoskeleton remodeling [34,35]. Binding of activated Rac to Pak1 releases Pak1 from intramolecular inhibition and results in autophosphoryation at several residues, including Ser-199, Ser-204, and Thr-423, with Thr-423 phosphorylation being required for full kinase activation [36,37,38]. We therefore analyzed the activity of the Rac signaling pathway in our panel of lung cancer cells by measuring levels of phosphorylated Pak1 in glucose-depleted cells. We observed increased levels of phospho-Ser199/204 and phospho-Thr423 in the subset of cell lines that survived in the absence of glucose compared to cell lines that required glucose for survival (Figure 3A). These results suggest that the Rac signaling pathway is more active in glucose independent cells compared to glucose addicted cells.

To determine if Rac activity is required to activate macropinocytosis, we utilized a small molecule inhibitor of Rac, EHT 1864. EHT 1864 binds all Rac isoforms with high affinity and inhibits Rac-GTP formation [39,40]. To confirm that EHT 1864 inhibits Rac activity, we treated cells with the inhibitor and analyzed levels of phosphorylated Pak1. Indeed, there were decreased levels of Pak phosphorylation following EHT 1864 treatment (Figure S3A). We next pretreated glucose starved cells with EHT 1864 prior to incubation with FITC-dextran. This resulted in a dramatic decrease in macropinocytotic activity, supporting the role of Rac in the regulation of macropinocytosis (Figure 3B, Figure S3B). We also determined if decreasing macropinocytosis through Rac inhibition affected the viability of glucose starved cells. Following treatment with EHT 1864, we observed a significant reduction in cell survival under glucose-free conditions (Figure 3C). However, in the presence of full glucose media, neither EIPA nor EHT 1864 affected cell proliferation (Figure S3C), suggesting that Rac-mediated macropinocytosis of extracellular protein is critical during glucose starvation. To determine if Rac signaling was responsive to glucose withdrawal, we compared the levels of phosphorylated Pak and Rac in cells cultured with or without glucose. There was no significant change in Rac expression or Pak activation following glucose withdrawal, suggesting that Rac signaling is not stimulated by glucose withdrawal (Figure S4A). Similarly, macropinocytosis does not appear to be regulated by glucose, as dextran uptake remained higher in glucose independent cells compared to glucose addicted cells in the presence of glucose (Figure S4B).

### 2.4. Rac1 Expression Regulates Macropinocytosis in a Pak Dependent Manner and is Necessary for Glucose Independence

**Figure 4:**
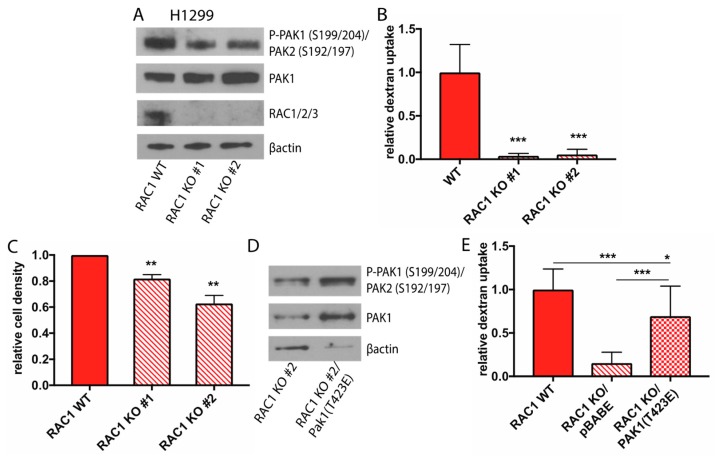
Rac1 expression activates macropinocytosis in H1299 cells in a PAK dependent manner and is necessary for glucose independence. (**A**) Immunoblot analysis comparing Rac signaling in Rac1 WT and Rac1 KO H1299 cells generated using CRISPR-Cas9 technology. The two knockout clones were generated using two independent guides against Rac1. (**B**) Quantification of the levels of FITC-dextran uptake in glucose starved H1299 Rac1 WT and H1299 Rac1 KO cells. Data is normalized to Rac1 WT cells, error bars indicate ± SD of 10 fields scored containing at least 10 cells. (**C**) Relative density of Rac1 WT and H1299 Rac1 KO cells cultured in GFM for 48 h as determined by crystal violet staining. Data is normalized to density of Rac1 WT cells at 48 h, error bars indicate ± SEM of three independent experiments. For (**B**,**C**), significance was calculated using Student’s *t*-test. (**D**) Immunoblot analysis of Pak signaling in Rac1 KO H1299 control cells or cells stably overexpressing the constitutive active Pak1 mutant T423E. (**E**) Quantification of the levels of FITC-dextran uptake in glucose starved Rac1 WT, Rac1 KO, and Rac1 KO/Pak1 (T423E) cells. Data is normalized to Rac1 WT cells; error bars indicate ± SD of 10 fields scored containing at least 10 cells. Significance was calculated using ANOVA with Holm-Sidak multiple comparisons across cell lines, * *p* < 0.05, ** *p* < 0.005, *** *p* < 0.0001.

To complement the pharmacological experiments using the Rac inhibitor, we utilized CRISPR/Cas9 mediated genome editing to delete the Rac1 isoform, which is ubiquitously expressed compared to Rac2 and Rac3, in two different glucose independent cell lines, H1299 and H1975. Two guide RNAs against *Rac1* were used to generate two knockout (KO) clones per cell line. Knockout genotypes were determined by western blot analysis of individual isolated clones. In addition to loss of Rac1 protein, Rac1 KO cells had reduced levels of phosphorylated Pak, suggesting that Pak activation is dependent on Rac1 expression in these cells (Figure 4A, Figure S5A). The levels of macropinocytosis in Rac1 wild-type (WT) and Rac1 KO cells was determined by fluorescence microscopy following incubation with FITC-dextran. Loss of Rac1 expression led to a significant decrease in levels of macropinocytosis in both H1975 and H1299 cells (Figure 4B, Figures S5B and S6A). In addition, a significant decrease in cell viability was observed in Rac1 KO cells when cultured in the absence of glucose, while proliferation rates were comparable in full glucose media (Figure 4C, Figure S5C,D).

We hypothesized that cells undergoing high rates of macropinocytosis may be dependent on the metabolism of extracellular protein for in vivo tumor growth. Therefore, we performed xenograft experiments using H1299 and H1975 Rac1 WT and KO cells to determine if loss of Rac signaling and macropinocytosis affects tumor formation. While the proliferation rate of Rac1 WT and Rac1 KO cells were equivalent in the presence of glucose when grown in 2D culture (Figure S5D), only cells expressing Rac1 were capable of forming tumors in vivo (Figure S7). Specifically, 5/5 (H1299) and 10/10 (H1975) mice injected with Rac1 WT cells developed tumors, while 0/10 (H1299) and only 3/20 (H1975) Rac1 KO xenografts formed small masses. These results suggest that cancer cells that undergo high levels of macropinocytosis are reliant on Rac1 expression for tumor formation. These results corroborate those using the Rac inhibitor and identify Rac signaling as a critical regulator of macropinocytosis and glucose free survival.

We have shown that the level of active, phosphorylated Pak is increased in glucose independent cells. Therefore, we wished to determine whether activation of Pak alone is sufficient to drive macropinocytosis downstream of Rac activation. To examine this, we expressed a constitutively active Pak1 mutant, Pak1 (T423E), in Rac1 KO H1299 and H1975 cells to see if this would restore macropinocytosis. Expression of this mutant led to an increase in Pak activity as determined by autophosphorylation (Figure 4D, Figure S5E). Expression of Pak1 (T423E) also resulted in a more than four-fold increase in dextran uptake compared to Rac1 KO cells (Figure 4E, Figures S5F and S6B), suggesting that Pak is a critical downstream Rac target that regulates macropinocytosis.

### 2.5. PI3K Regulates Rac-Driven Macropinocytosis

**Figure 5:**
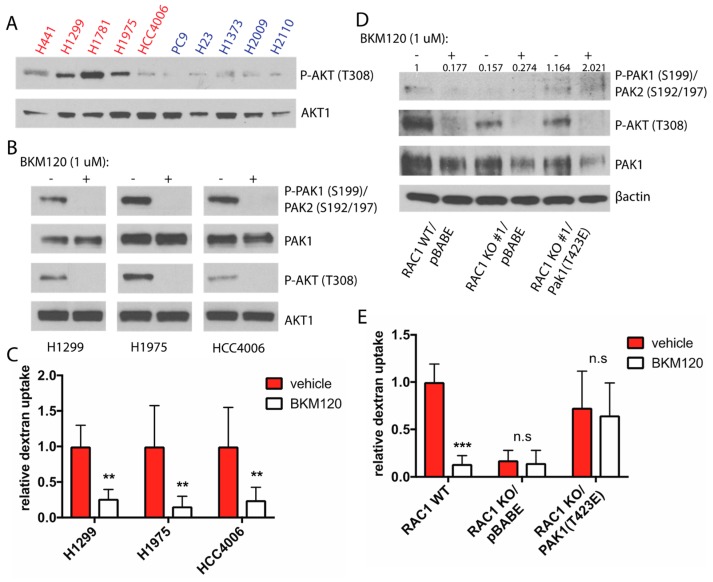
PI3K regulates Rac-driven macropinocytosis. (**A**) Immunoblot analysis of phospho-Akt (Thr308) in glucose independent (red) and glucose addicted (blue) cells following 6 h of glucose starvation. (**B**) Immunoblot analysis of Pak and Akt phosphorylation following treatment with vehicle or 1 μm BKM120 for 1 h. (**C**) Quantification of FITC-dextran uptake in glucose starved cells treated with either vehicle or 1 μm BKM120 for 1 h prior to incubation with FITC-dextran. Data is normalized to vehicle treated cells. Error bars indicate ± SD of 10 fields scored containing at least 10 cells, and statistics was calculated using Student’s *t*-test. (**D**) Immunoblot analysis of Pak signaling in Rac1 KO H1299 control cells or cells stably overexpressing the constitutive active Pak1 mutant T423E following treatment with vehicle or 1 μm BKM120 for 1 h. Densitometry was used to quantify P-Pak band intensities normalized to T-Pak1. (**E**) Quantification of FITC-dextran uptake in glucose starved Rac1 WT, Rac1 KO, and Rac1 KO/Pak1 (T423E) cells treated with vehicle or 1 μm BKM120 1 h prior to incubation with FITC-dextran. Data is normalized to Rac1 WT cells, error bars indicate ± SD of 10 fields scored containing at least 10 cells. Significance was calculated using ANOVA with Holm-Sidak multiple comparisons between control and BKM120 treated cell lines, ** *p* < 0.005, *** *p* < 0.0001.

We have shown that the Rac-Pak signaling cascade is a crucial activator of macropinocytosis in NCSLC cells, yet it is important to identify the upstream activator of this pathway in order to shed further light on how macropinocytosis and glucose independent tumor growth might be targeted. Activation of Rac occurs through interactions with guanine nucleotide exchange factors (GEFs), which catalyze the dissociation of Rac from GDP, thereby allowing GTP to bind and activate Rac. Several Rac-GEFs, including P-Rex1, P-Rex2, Sos, Vav, and Tiam are activated by binding to the second messenger phosphoatidylinositol-3,4,5-trisphosphate (PIP3), which is generated by phosphoinositide-3 kinase (PI3K) [41]. Interestingly, we observed higher levels of Thr-308 phosphorylated Akt (a PIP3 dependent Ser/Thr kinase) in glucose independent cell lines (Figure 5A), implying that these cells have elevated PI3K signaling. To understand if PI3K regulates Rac signaling in glucose independent cells, we incubated cells with the pan-class I PI3K inhibitor BKM120 and analyzed levels of phosphorylated Pak. Treatment with BKM120 inhibited activation of Akt as measured by phosphorylation at Thr-308, and also resulted in a concomitant decrease in phospho-Pak (Figure 5B). To examine the effect of BKM120 treatment on macropinocytosis, we pre-treated glucose starved cells with BKM120 prior to incubation with FITC-dextran. Macropinocytosis was significantly decreased in the cell lines tested (Figure 5C, Figure S8A).

We next examined if the observed inhibition of macropinocytosis in response to BKM120 treatment was a result of decreased Pak activity. To do so, we utilized our H1975 Rac1 KO cells stably expressing highly active Pak1 (T423E). Phosphorylation of Pak was decreased in Rac1 WT cells following incubation with BKM120, while Pak phosphorylation was unaffected in Rac1 KO cells expressing Pak1 (T423E) (Figure 5D). We next analyzed levels of macropinocytosis in these cells, and found that while treatment of Rac1 WT cells with BKM120 resulted in decreased macropinocytosis, there was no difference in levels of macropinocytosis in Rac1 KO/Pak1 (T423E) cells treated with PI3K inhibitor (Figure 5E, Figure S8B). These results identify PI3K as an upstream activator of Rac-Pak signaling and macropinocytosis.

### 2.6. Internalized Protein Supplies Glucose Starved Cells with Alanine and Other Free Amino Acids

**Figure 6:**
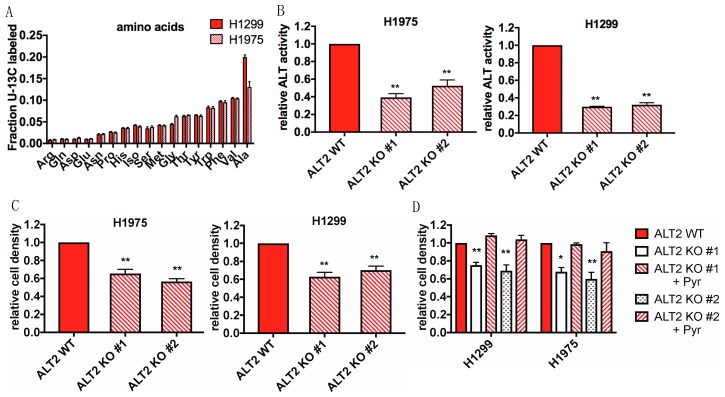
Alanine is derived from internalized protein and is converted to pyruvate by ALT2 to support proliferation during glucose starvation. (**A**) Detection of universally (U) ^13^C-labeled amino acids in cells cultured in GFM supplemented with unlabeled or U-^13^C labeled yeast protein by LC-MS. Values are presented as a ratio of U-^13^C labeled amino acid to total amino acid abundance, Error bars indicate ± SD of three biological replicates. (**B**) Relative ALT activity of ALT2 WT and ALT2 KO H1299 and H1975 cell lysates. Data is normalized to ALT2 WT cells. (**C**) Relative density of ALT2 WT and ALT2 KO H1299 and H1975 cells cultured in GFM for 48 h. Data is normalized to density of ALT2 WT cells at 48 h. For (**B**,**C**), Significance was calculated using Student’s *t*-test. (**D**) Relative density of glucose starved ALT2 WT and ALT2 KO H1299 and H1975 cells supplemented with vehicle or 1 mM pyruvate (Pyr). Data is normalized to density of vehicle treated ALT2 WT cells at 48 h. Significance was calculated using ANOVA with Holm-Sidak multiple comparisons to ALT WT cell lines. For (**B**–**D**), error bars indicate ± SEM of at least three independent experiments, * *p* < 0.05, ** *p* < 0.005.

We next wished to determine if internalized protein could supply glucose starved cells with free U-^13^C glucose and U-^13^C amino acids to trace incorporation of labeled carbon into intracellular amino acids. H1299 and H1975 cells were cultured for 24 h in media lacking both glucose and serum and supplemented with either unlabeled or ^13^C labeled yeast protein. We confirmed the uptake of yeast protein by observing co-localization of rhodamine-labeled yeast protein and FITC-dextran in cells (Figure S9). Following incubation with unlabeled or ^13^C labeled protein, intracellular metabolites were extracted and quantified by liquid chromatography/mass spectrometry. We detected significant quantities of uniformly ^13^C labeled amino acids in H1975 and H1299 cells (Figure 6A). Interestingly, the amino acid with the highest fraction of ^13^C incorporation was alanine. We therefore traced the metabolic fate of alanine during glucose starvation by culturing H1299 and H1975 cells in glucose depleted media supplemented with unlabeled or uniformly ^13^C-labeled alanine. There was significant ^13^C labeling of TCA intermediates including citrate, α-ketoglutarate, succinate, and malate, as well as glycolytic intermediates 3-phosphoglycerate (3-PG) and lactate (Figure S10A). The predominance of M+2 labeled TCA cycle intermediates implies that pyruvate is generated from alanine via the enzyme alanine transaminase (ALT), which can then be converted to acetyl CoA by pyruvate dehydrogenase to feed the TCA cycle. The presence of M+1 and M+2 3-PG suggests that it is derived from phosphoenolpyruvate that was formed via decarboxylation of oxaloacetate by PEPCK (Figure S10B). Taken together, our data shows that alanine produced from the degradation of internalized protein can support cell growth during glucose withdrawal by supplying the cell with a source of pyruvate to feed the TCA cycle and gluconeogenesis.

### 2.7. ALT2 Activity is Critical for Proliferation During Glucose Starvation

Alanine transaminase catalyzes the reversible transamination between alanine and α-ketoglutarate to form pyruvate and glutamate. There are two different ALT isoforms- ALT1 which is cytosolic and ALT2 which is mitochondrial [42]. Recent studies have uncovered important roles for ALT2 in adaptive cancer metabolism [43,44,45,46,47]. Analysis of expression data from the Cancer Cell Line Encyclopedia (CCLE) revealed that *ALT2* expression is significantly higher than *ALT1* in various lung cancer cell lines (Figure S11A). Furthermore, we found that ALT2 protein was expressed in both H1299 and H1975 cells, while ALT1 was not expressed in H1975 cells (Figure S11B). Despite lacking ALT1, H1975 cells had alanine transaminase activity that was comparable to H1299 cells (Figure S11C). We therefore deleted ALT2 in H1975 and H1299 cells using CRISPR-Cas9 technology in order to evaluate its role in protein-fueled glucose independence (Figure S11D). Loss of ALT2 expression resulted in a significant decrease in transaminase activity of both H1299 and H1975 cell lysates, suggesting that it is critical for the conversion of alanine to pyruvate (Figure 6B). To determine if ALT2 activity is critical for survival during glucose starvation, we cultured ALT2 WT and ALT2 KO cells in glucose free media. Indeed, ALT2 KO cells were significantly more sensitive to glucose withdrawal compared to ALT2 WT cells, while ALT2 WT and ALT2 KO cells had comparable growth under glucose-replete conditions. (Figure 6C, Figure S11E). Furthermore, supplementation with exogenous pyruvate rescued the viability of ALT2 KO cells, suggesting that pyruvate produced from alanine supports cell survival in the absence of glucose (Figure 6D). To corroborate the results seen with ALT2 knockout cells, we also treated cells with the ALT inhibitor L-cycloserine (L-CS). L-CS treatment, as well as boiling to destroy all enzyme activity, significantly reduced the transaminase activity of both cell lines (Figure S11C). In agreement with the results seen with ALT2 KO cells, treatment with L-CS resulted in decreased cell survival under conditions of glucose deprivation (Figure S11F). Interestingly, supplementing glucose and serum free media with exogenous alanine only partially rescued the viability of glucose independent cells. (Figure S11G), suggesting that while alanine is necessary for glucose free survival, the internalization of other amino acids is also required. Overall, these data show that the transamination of alanine to pyruvate by ALT2 is required for metabolic adaptation to glucose withdrawal.

## 3. Discussion

Targeting cancer cell metabolism has emerged as an attractive potential therapeutic avenue. Tumor cells, unlike normal undifferentiated cells, have a high demand for energy and biomass in order to fuel their rapid growth, and the flexibility of tumor metabolism is important for survival in nutrient poor tumor microenvironments. We therefore set out to identify adaptive metabolic pathways utilized by cancer cells during glucose starvation and potentially uncover novel drug targets. Here, we found that NSCLC cells scavenge extracellular protein in the absence of glucose via Rac-mediated macropinocytosis, which supplies cells with amino acids required for growth.

Previous studies have also identified macropinocytosis as a critical process for cancer cell survival in nutrient poor environments. In Ras mutant pancreatic and bladder cancer cells, macropinocytosis of extracellular protein has been shown to be dependent on oncogenic Ras expression and necessary for cell growth during glutamine starvation. Furthermore, inhibiting macropinocytosis leads to a reduction in pancreatic xenograft tumor growth in vivo [26]. Another study reported that human pancreatic ductal carcinoma tumor samples, which are poorly vascularized and whose microenvironment has decreased levels of metabolites including glucose, undergo high rates of macropinocytosis to maintain intracellular amino acid levels [5]. Furthermore, the internalization of labeled albumin and its subsequent breakdown into amino acids was directly observed in pancreatic tumors of live *Kras^G12D^;p53^−/−^* mice by live microscopy [27]. In addition to protein internalization, it has also been shown that consumption of extracellular ATP by lung cancer cells via macropinocytosis increases intracellular ATP levels and protects cells from inhibition of glycolysis and oxidative phosphorylation [48]. Therefore, it is possible that the uptake of other soluble components in the tumor microenvironment in combination with internalized amino acids can supply the cell with the necessary metabolites to fuel various biosynthetic processes required for proliferation.

While we found no clear genetic basis for glucose independence, we demonstrated that Rac signaling and activation of the effector protein Pak is increased in glucose independent cells and is critical for macropinocytosis and glucose free survival. Specifically, both inhibition of Rac using a small molecule inhibitor and genetic deletion of Rac1 resulted in decreased macropinocytosis and sensitization to glucose starvation. The reduction in macropinocytosis was rescued by expression of a constitutive active Pak1 mutant. Furthermore, we identified PI3K as the critical upstream activator of Rac-mediated macropinocytosis. Of note, Rac-Pak signaling in the glucose independent cell line HCC4006 was relatively low compared to the other glucose independent cell lines, yet macropinocytosis activity was high. While our experiments using Rac and PI3K inhibitors suggest that the Rac-Pak signaling pathway is involved in part in regulating macropinocytosis and glucose free survival, it is interesting to note that HCC4006 cells have an activating ELR746del mutation and amplification of EGFR. Phospholipase C is a downstream target of EGFR, which is also known to activate macropinocytosis [33]. Therefore, in HCC4006 cells, co-activation of Rac and PLC by PI3K and EGFR may cooperate to fully activate macropinocytosis.

The role of Rac signaling in stimulating macropinosome formation is supported by previous studies performed in dendritic cells which show that constitutive macropinocytosis is inhibited by expression of dominant-negative Rac or treatment with a PI3K inhibitor [32]. It has also been reported that PI3K activates Rac-mediated, growth factor-induced macropinocytosis in fibroblasts, and expression of oncogenic PI3K or loss of PTEN increases the macropinocytotic capacity of fibroblasts and fuels cell growth in the absence of leucine [33]. Interestingly, tumor associated activating mutations in Rac1 and Rac2 have been identified in melanoma, sarcoma, and tumors of the head and neck, breast, and brain [49,50,51,52,53,54]. In addition, various Rac isoforms have been shown to be overexpressed in human cancers including lung, gastric, testicular, and breast [53,55,56,57,58]. We are interested in determining if the presence of activating Rac mutations and/or overexpression is a predictor of increased macropinocytosis and resistance to glucose withdrawal across tumor types.

By generating universally ^13^C yeast protein lysate, we were able to trace the flux of internalized protein into amino acids, and found significant labeling of alanine. Alanine generated TCA cycle and gluconeogenic intermediates, and conversion of alanine to pyruvate by ALT2 was critical for glucose free survival. Previous studies have shown that ALT2 is critical for the growth of certain tumors, and ALT2 expression is often regulated by different oncogenic alterations. In human breast cancer, tumor grade and proliferation correlates with an increase in ALT2 expression [43]. Furthermore, *PIK3CA* mutant colon cancer cells are reported to upregulate ALT2 expression, and inhibition of ALT2 activity decreases the growth of *PI3KCA* mutant tumors [45]. Colon cancer cells expressing oncogenic p53 and Ras also cooperate to upregulate expression of metabolic enzymes including ALT2. This oncogenic upregulation of ALT2 increases glutamate conversion to α-ketoglutarate to drive the TCA cycle [47]. In addition, alanine secreted by pancreatic stellate cells has been reported to be utilized by PDAC cells to fuel the TCA cycle in an ALT dependent manner [46].

We found that while supplementing glucose and serum free media with exogenous alanine increased cell viability, it was not a complete rescue, as compared to albumin supplementation. An explanation for this is that amino acids derived from internalized proteins are needed to fuel the TCA cycle, allowing alanine-derived pyruvate to be used primarily to produce glycolytic intermediates. Furthermore, unidirectional amino acid uptake into cancer cells from the culture media or into tumors from blood is relatively slow. Thus, amino acids derived from degradation of internalized proteins not only provide a rapid source for new protein synthesis, but also provide a currency of exchange for extracellular amino acids needed for other anabolic processes [59,60]. Collectively, our results show that targeting either Rac-driven macropinocytosis or ALT2 mediated pyruvate production may have therapeutic implications for the treatment of NSCLC.

## 4. Materials and Methods

### 4.1. Reagents and Plasmids

Antibodies were purchased from the following suppliers: Cell Signaling Technologies (Danvers, MA, USA) (phospho Pak1 S199/204Pak2 S192/197-2605, phospho-Pak1 T423/Pak2 T402-2601, Pak1-2602, Rac1/2/3-2465, phospho Akt Thr308-4056, Akt1-2938) Abcam (Cambridge, MA, USA) (βactin-ab7817) and Santa Cruz Biotech. (Santa Cruz, CA, USA) (ALT1-S374501, ALT2-398383). Inhibitors and metabolites were purchased from Selleck Chemicals (Houston, TX, USA) (BKM120), Sigma-Aldrich (St. Louis, MO, USA) (EIPA, L-cycloserine, L-alanine, pyruvate), and Santa Cruz Biotech. (EHT 1864). PAK1 T423E cDNA was obtained from pCMV6M-PAK1 T423E, a gift from Jonathan Chernoff purchased from Addgene (Addgene plasmid #12208, Watertown, MA, USA). The PAK1 T423E cDNA was sub-cloned into the retroviral vector pBABE-puro for stable expression via retroviral infection.

### 4.2. Cell Culture

NSCLC cell lines were obtained from the Hamon Cancer Center Collection (University of Texas–Southwestern Medical Center, Dallas, TX, USA). All cell lines were maintained under 5% CO_2_ at 37 °C in RPMI-1640 medium (Corning, 10040, Corning, NY, USA) supplemented with 10% FBS (Denville, 5002, Metuchen, NJ, USA) and 100 μl/mL penicillin and 100 μg/mL streptomycin (Gibco, 15140122, Gaithersburg, MD, USA). For glucose starvation experiments, cells were cultured in glucose-free media (GFM) containing RPMI 1640 (Thermo Fisher Sci.,11879-020, San Jose, CA, USA) supplemented with 10% dialyzed FBS (Gibco, 26400044) or 2% (*w*/*v*) Fraction V BSA (EMD Millipore, 126609, Burlington, MA, USA).

### 4.3. Cell Line Generation

To generate Rac1 knockout cell lines, two different 20-bp guide sequences targeting Rac1 (5′-GTGTGAGCGCCGAGCACTCC-3′) and (5′-TTACTGTTTGCGGATAGGAT-3′) were chosen using a publicly available online CRISPR guide design tool at http://crispr.mit.edu [61]. To generate ALT2 knockout cells, a guide containing the sequence (5′-CAGCTCGAGCTCGATCTCGC-3′) was used. The guide sequences were cloned into PX459 V2.0, a gift from Feng Zhang purchased from Addgene (Addgene, #62988). Cell lines were transfected with guide RNA plasmids using Lipofectamine LTX (Thermo Fisher Sci., 15338100), and selected with puromycin at 1.5 μg/mL 24 h post transfection. Single cells were plated at low density in 10 cm. plates, and single colonies were collected after 2 weeks. Knockout clones were screened by western blotting.

### 4.4. Generation of Retroviral PAK (T423E) Stable Cell Lines

Retrovirus was produced by transfection of pBabe constructs into the Phoenix HEK293 packaging cell line (ATCC) using Lipofectamine 2000 (Thermo Fisher Sci., 11668027). Virus was collected 24 and 48 h after transfection, and passed through a 0.45 μm PVDF membrane filter. For infection of cell lines, polybrene (Santa Cruz, SC-134220) was added to the virus at a concentration of 8 μg/mL and added to cells plated at 20% confluency for 6 h. Fresh media was then added, and cells were selected with 1.5 μg/mL puromycin 48 h after the initial point of infection. Cells were maintained in selection media for 1 week following complete selection of control, uninfected cells.

### 4.5. Xenograft Assays

FoxN1 female nude mice at 7 weeks of age were injected subcutaneously into the fat pad with 1 × 10^6^ cells in a 1:1 mixture of growth media and Matrigel (Corning, 354230). Tumor size was recorded using calipers, and tumor volume was calculated using the formula 4/3 × π × R^3^, where R is √(width × height)/2. Mice were sacrificed when tumors reached a diameter of 1.5 cm. Animal studies were approved by the IACUC as described in protocol #2013-0116, which was most recently renewed on 20 September 2018.

### 4.6. Immunoblotting

Cells were washed with ice-cold PBS, and scraped following addition of lysis buffer (150 mM NaCl, 25 mM Tris pH 7.4, 0.1% Triton X-100, 1 mM EDTA). 1× Laemmli buffer (125 mM Tris pH 6.8, 10% (*v*/*v*) 2-mercaptoethanol, 4% (*w*/*v*) SDS, 20% (*v*/*v*) glycerol, 0.05% bromophenol blue) was added and samples were boiled for 5 min. Samples were separated by SDS/PAGE on Tris-glycine gels (Thermo Fisher Sci., XP04200BOX) and transferred onto nitrocellulose (Bio-Rad, 1620115, Hercules, CA, USA). Membranes were blocked with 5% (*w*/*v*) nonfat milk in Tris-buffered saline and Tween 20 buffer (TBST) and incubated with the appropriate antibody overnight at 4 °C. Membranes were washed three times with TBST and the appropriate secondary antibody was added for 1 h at room temperature. Blots were developed using ECL (Thermo Fisher Sci., 34076) and audioradiography film (Denville, E3018).

### 4.7. Glucose-Free Growth Assays

To measure total viable cell content, a total of 15,000 cells were plated per well in a 48-well plate in triplicate. After 24 h, cells were rinsed and incubated with glucose free media (GFM). Cells were incubated for 48 h, and then collected to measure cell density. A day 0 plate was also collected. To measure cell density, cells were fixed and stained with 0.05% crystal violet in 10% formalin for 20 min. Each well was then washed multiple times with PBS. For quantification of cell density, crystal violet stain was resolubilized in 10% acetic acid and the absorbance at 595 nm was recorded using a Fluostar Omega plate spectrophotometer. Change in cell density was calculated by normalizing absorbance at a given time point to the absorbance at day 0. For relative cell density, absorbance was normalized to the absorbance of a control cell line as indicated.

### 4.8. Macropinocytosis Visualization and Quantification

Macropinocytosis quantification was adapted from a previously described protocol [62]. Cells were plated in 24 well plates containing glass coverslips coated with Poly-D-lysine (Trevigen, Gaithersburg, MD, USA). After 24 h of attachment, cells were incubated in glucose-free medium (GFM) for 3 h, followed by addition of 1 mg/mL FITC-dextran, MW 70,000 (Thermo Fisher Sci., D-1822) for 1 h. Cells were washed with ice-cold phosphate buffered saline PBS 4 times, followed by one wash with a low pH buffer (0.1 M sodium acetate, 0.05 M NaCl, pH 5.5) to bleach surface bound dextran. Cells were then fixed with 3.7% formaldehyde in PBS for 30 min. at room temperature, and then washed twice with PBS. Cells were stained with 5 μg/mL Wheat Germ Agglutinin, Alexa Fluor 594 Conjugate, and washed once more (Thermo Fisher Sci. W1126). Cells were mounted on glass slides with ProLong Diamond Antifade Mountant with DAPI (Life Tech., P3696, Carlsbad, CA, USA) and incubated in the dark overnight. Images were acquired with a Nikon Eclipse Ti-E inverted microscope. Uptake of fluorescently labeled dextran was quantified using the Nikon analysis software. Wheat germ agglutinin fluorescence was used to detect and select cells using the region of interest auto detect function. An intensity threshold for dextran fluorescence was determined, and the object count function was used to calculate the percent area of internalized fluorescent dextran normalized to the total area of cells. This value was then normalized to a control cell line to generate the value named “relative dextran uptake”.

### 4.9. Generation of ^13^C-Labeled Proteins

A haploid SK1 strain of Saccharomyces cereviseae was grown to an OD of 1.0 in Difco yeast nitrogen base lacking amino acids (BD, Franklin Lakes, NJ, USA, 291940, Detroit, MI, USA) supplemented with 2% normal isotopic (Sigma, G270) or U-^13^C labeled glucose (Cambridge Isotope Lab., CLM-1396-25, Tewksbury, MA, USA) and 1X unlabeled or U-^13^C labeled Bioexpress cell growth media (Cambridge Isotope Lab., CGM-1000-U-0, CGM-1000-C-0). Cells were washed twice with water, and once with urea lysis buffer (8 M urea, 50 mM Tris, pH 7.5, 50 mM NaCl, 1 mM EDTA, 2 mM MgCl_2_, protease inhibitor tablets (Thermo Fisher Sci, A32959). Cell pellets were lysed with 1 g acid washed beads per gram cell weight in 1 mL urea lysis buffer per gram cell weight and vortexed for 1 min, 5 times. Lysate was spun down at 10,000× *g* for 10 min. to remove beads, and then again at 20,000 × g for 30 min. to clarify lysate. Lysate was diluted 1:1 with 50 mm Tris-HCl, pH 7.5, 50 mM NaCl, 2 mM MgCl_2_, concentrated using a Pierce 10K protein concentrator (Thermo Fisher Sci., 88517), and treated with 2500U Benzonase (EMD Millipore, 70664-3,) at 37 °C for 1 h. Samples were dialyzed overnight against PBS using 10K MW dialysis cassettes (Thermo Fisher Sci., 66003). The protein was then either tagged with NHS-Rhodamine using the instructions provided by the Pierce NHS-Rhodamine labeling kit (Thermo Fisher Sci., 53031), or used to supplement glucose-free media for metabolite tracing experiments.

### 4.10. Metabolic Tracing and Extraction

Cells were plated in triplicate in 6 well plates. When cells reached 80% confluency, they were washed with PBS and incubated with glucose-free RPMI supplemented with 0.75 mg/mL purified unlabeled or ^13^C labeled yeast protein or 1 mM unlabeled or U-^13^C labeled L-alanine for 24 h. To extract intracellular metabolites, cells were washed with ice cold PBS, and quenched with 1 mL ice cold 80% methanol. Extracts were clarified by centrifugation at 14,000 rpm for 10 min, and the metabolites were dried using a Speed-Vac Evaporator.

### 4.11. Liquid Chromatography/Mass Spectrometry (LC/MS) Analysis

Metabolomics analysis was performed as described previously [63]. Briefly, metabolites were extracted from cells using precooled 80% methanol. LC/MS analyses were performed on a Q Exactive Orbitrap mass spectrometer (Thermo Fisher Sci.) coupled to a Vanquish UPLC system (Thermo Fisher Sci.). The Q Exactive operated in polarity-switching mode. A Sequant ZIC-HILIC column (2.1 mm i.d. × 150 mm, Merck) was used for separation of metabolites. Flow rate was set at 150 μL/min. Buffers consisted of 100% acetonitrile for mobile A, and 0.1% NH_4_OH/20 mM CH_3_COONH_4_ in water for mobile B. Gradient ran from 85% to 30% A in 20 min followed by a wash with 30% A and re-equilibration at 85% A. Metabolites and their ^13^C isotopologues were identified on the basis of exact mass within 5 ppm and standard retention times. Relative metabolite quantitation was performed based on peak area for each metabolite. All data analysis was done using in-house written scripts.

### 4.12. ALT Activity Assay

ALT activity was measured using a commercially available kit (BioVision, K752-100, Milpitas, CA, USA). 1 × 10^6^ cells were homogenized in 200 µL ice cold ALT assay buffer, and centrifuged at 13,000× *g* to remove insoluble material. Samples were then boiled for 5 min or treated with 250 µM L-cycloserine for 15 min. A reaction mixture containing ALT enzyme mix, ALT substrate, and fluorescent peroxidase substrate was added to lysates per the manufacturer’s protocol. Fluorescence were measured at 37 °C every 5 min. at 570 nm. Standard curves were generated, and ALT activity was calculated using the equation: ALT. Activity = nmole of pyruvate generated/(Time_final_–Time_initial_) × Volume (mL). ALT activity was reported as milliunit/mL, where one milliunit (mU) of ALT is the amount of enzyme needed to generate 1.0 nmole of pyruvate per minute at 37 °C.

### 4.13. Public Dataset: Cancer Cell Line Encyclopedia (CCLE) Data Analysis

The expression dataset of ALT1 and ALT2 was accessed and downloaded at http://www.broadinstitute.org/ccle/home.

### 4.14. Statistical Analysis

*P* values were calculated by unpaired Student’s *t*-test.

## 5. Conclusions

Here, we show that NSCLC cells can survive and proliferate under glucose depleted conditions and rely on the macropinocytosis of extracellular protein for fuel. These glucose independent cells have increased signaling through the Rac-Pak pathway, and activation of this pathway is essential for macropinocytosis and glucose-free survival. Furthermore, PI3K regulates macropinocytosis in a Pak dependent manner, and Rac expression is required for tumor formation in vivo. Internalized protein is degraded in the cell to produce free amino acids, including alanine, which generate TCA cycle and gluconeogenic intermediates. Expression of the mitochondrial enzyme ALT2, which converts alanine to pyruvate, is critical for glucose independence. Overall, these results uncover an adaptive metabolic pathway used by NSCLC cells to survive states of glucose deprivation.

## Supplementary Materials

The following are available online at http://www.mdpi.com/2072-6694/11/1/37/s1, Figure S1: FITC-dextran uptake in glucose independent and glucose addicted cells., Figure S2: Inhibition of macropinocytosis by EIPA, Figure S3: Inhibition of macropinocytosis by EHT 1864, Figure S4: Rac signaling and macropinocytosis is not regulated by glucose, Figure S5: Rac1 regulates macropinocytosis of H1975 cells in a PAK dependent manner to promote glucose independence, Figure S6: Rac1 activation of Pak regulates macropinocytosis, Figure S7: Rac1 is required for tumor formation of glucose independent cells, Figure S8: PI3K activates Rac-mediated macropinocytosis, Figure S9: Co-localization of internalized Rhodamine-labeled yeast protein (red) with FITC-dextran (green) puncta in H1975 cells, Figure S10: Alanine generates TCA cycle and gluconeogenic intermediates, Figure S11: ALT2 is expressed in NSCLC cells and converts alanine to pyruvate to support glucose free survival.
