# Supplementary material for: Rac-Mediated Macropinocytosis of Extracellular Protein Promotes Glucose Independence in Non-Small Cell Lung Cancer
Source: Cancers (Basel). 2019 Jan 2;11(1):37. doi: 10.3390/cancers11010037 (PMC6356657; doi:10.3390/cancers11010037)
Supplement: Supplementary file 1 [file cancers-11-00037-s001.pdf]

Supplementary Materials: Rac-Mediated Macropinocytosis of Extracellular Protein Promotes Glucose Independence in Non-Small Cell Lung Cancer

Cindy Hodakoski, Benjamin D. Hopkins, Guoan Zhang, Taojunfeng Su, Zhe Cheng, Roxanne Morris, Kyu Y. Rhee, Marcus D. Goncalves and Lewis C. Cantley

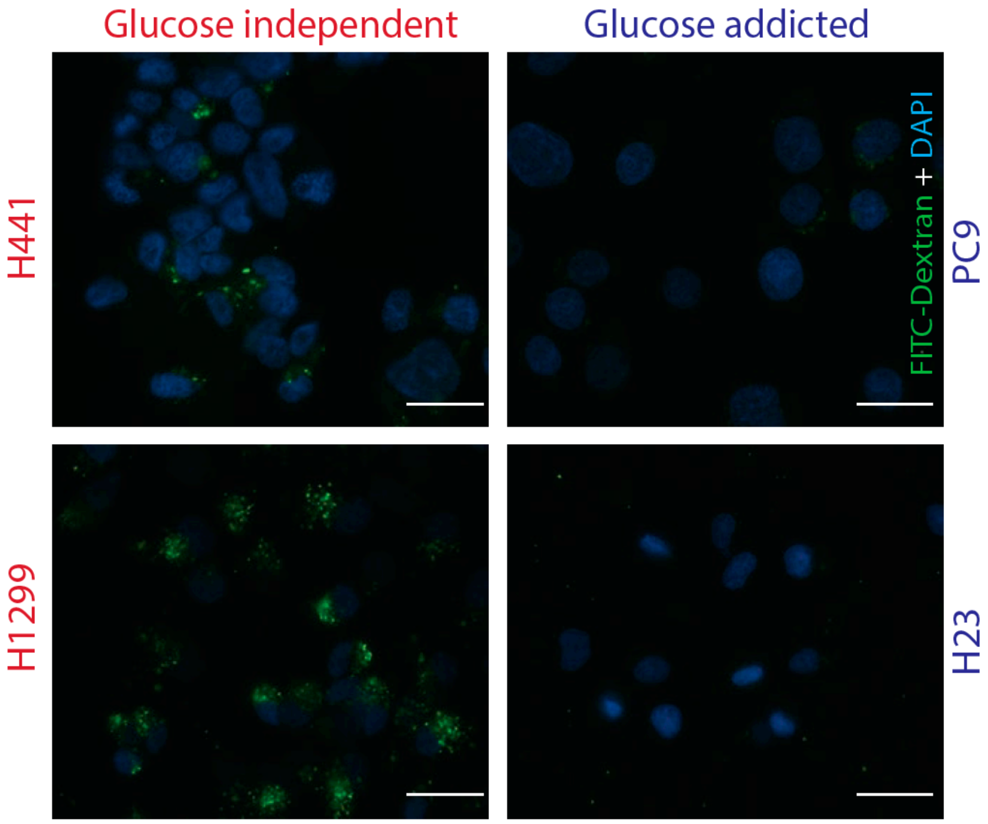

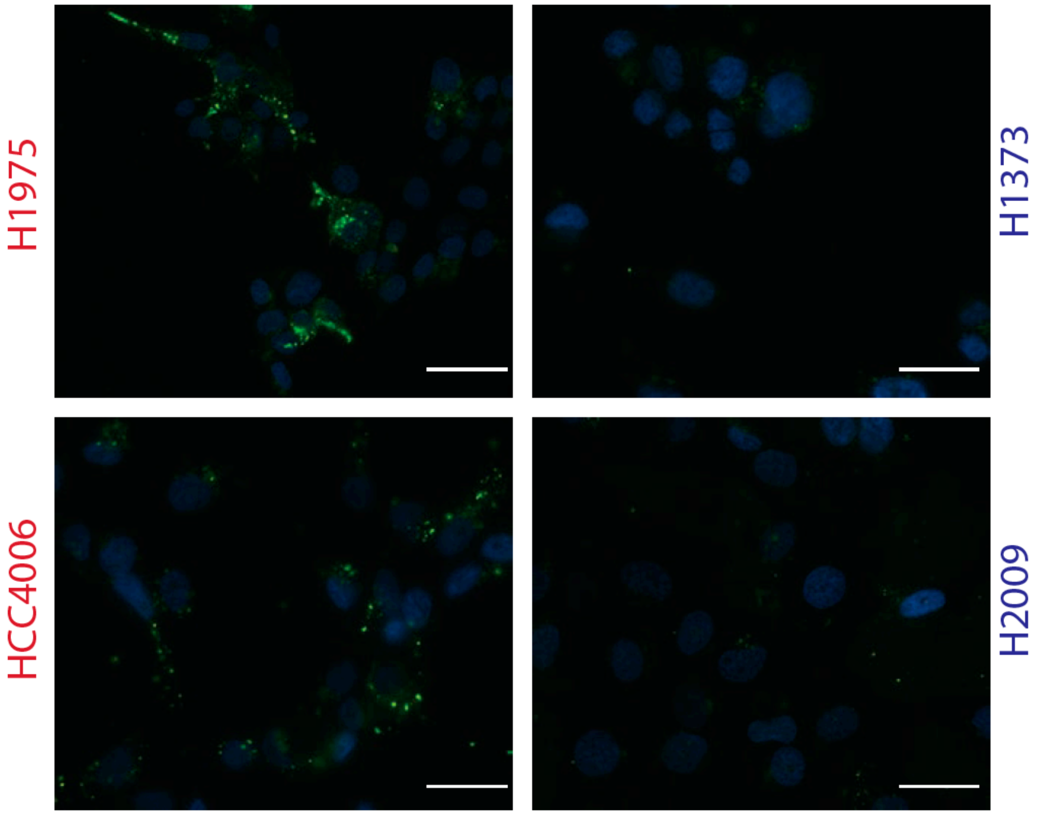

Figure S1. FITC-dextran uptake in glucose independent and glucose addicted cells. Representative images of FITC-dextran (green) internalization via macropinocytosis in NSCLC cells. Cells were cultured in glucose-free media (GFM) for 3 h prior to the addition of 1 mg/mL FITC-dextran for 1 h Scale bar, 50 µm.

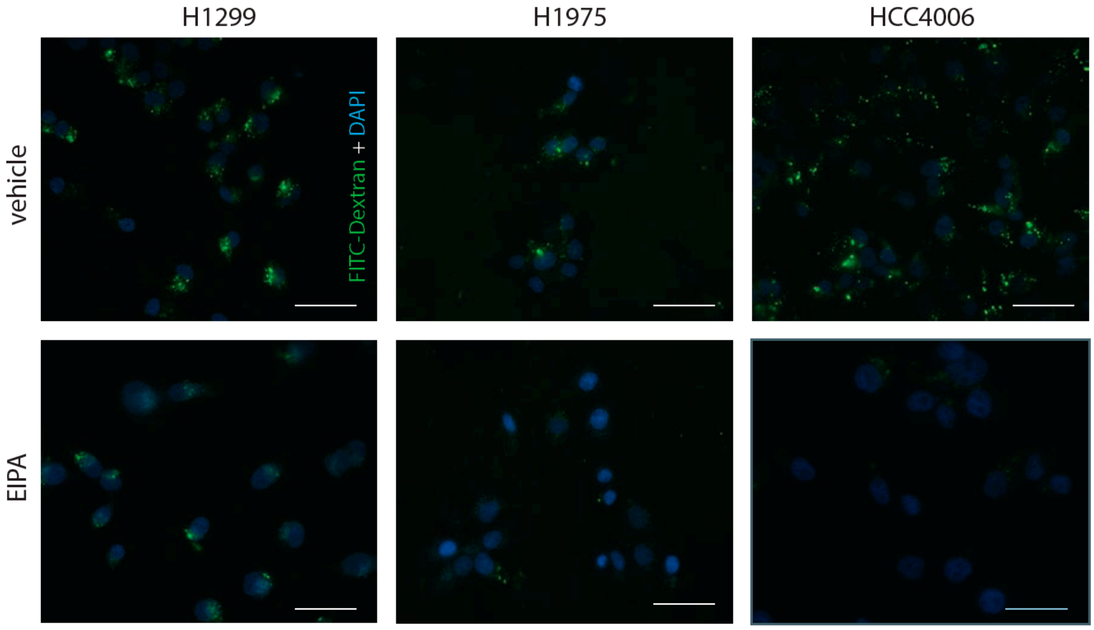

Figure S2. Inhibition of macropinocytosis by EIPA. Representative images of FITC-dextran (green) internalization in glucose starved glucose independent cells that were treated with vehicle or 10 µm EIPA 1 h prior to incubation with dextran. Scale bar, 50 µm.

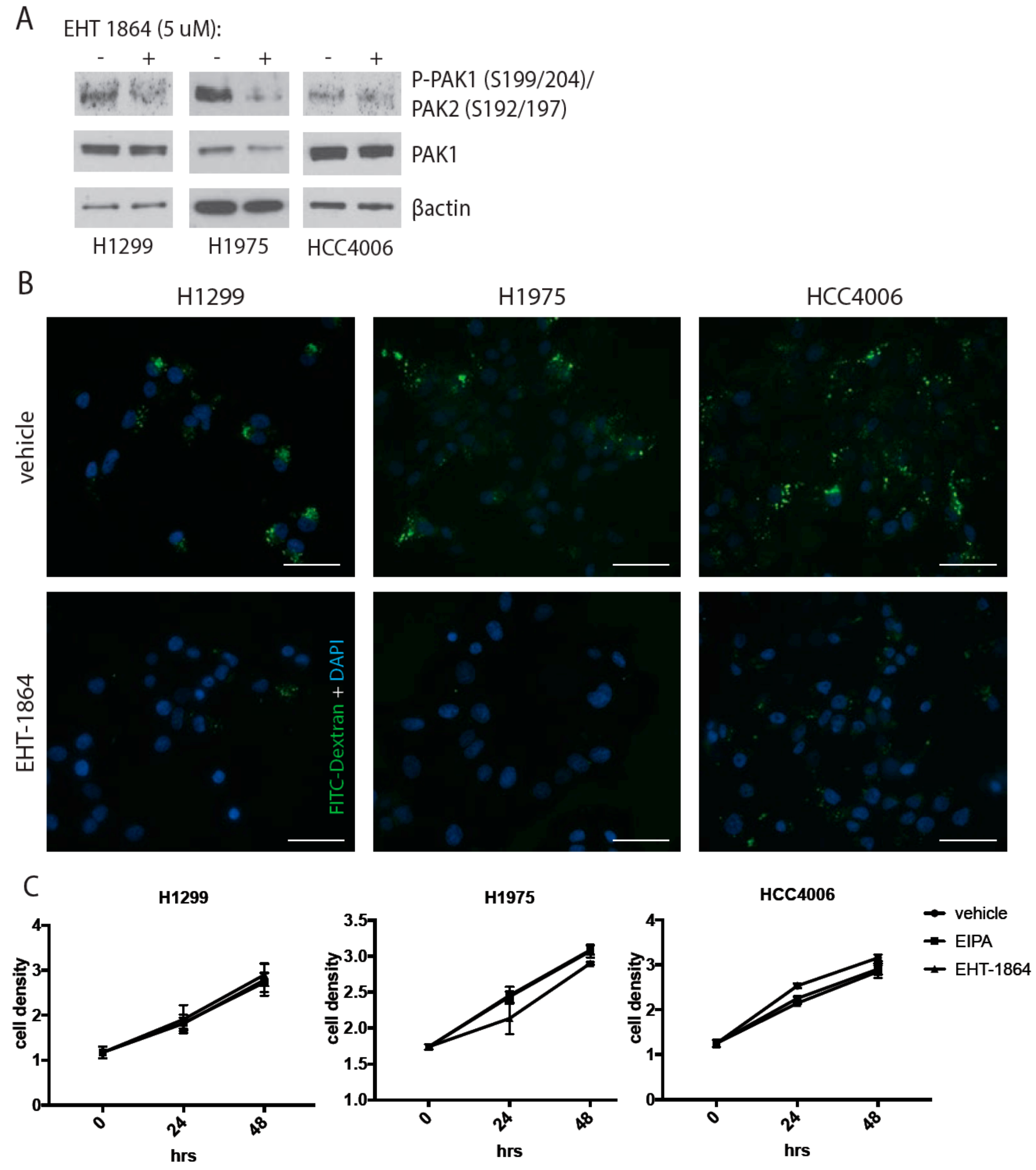

Figure S3. Inhibition of macropinocytosis by EHT 1864. (**A**) Immunoblot analysis of Pak phosphorylation following treatment with vehicle or 5 µm EHT 1864. (**B**) Representative images of FITC-dextran (green) internalization in glucose starved glucose independent cells treated with vehicle or 5 µm EHT 1864 1 h prior to incubation with dextran. Scale bar, 50 µm. (**C**) Density of cells treated with 10 µm EIPA or 5 µm EHT 1864 in full growth media. Error bars indicate +/- SD of three biological replicates, representative experiment shown.

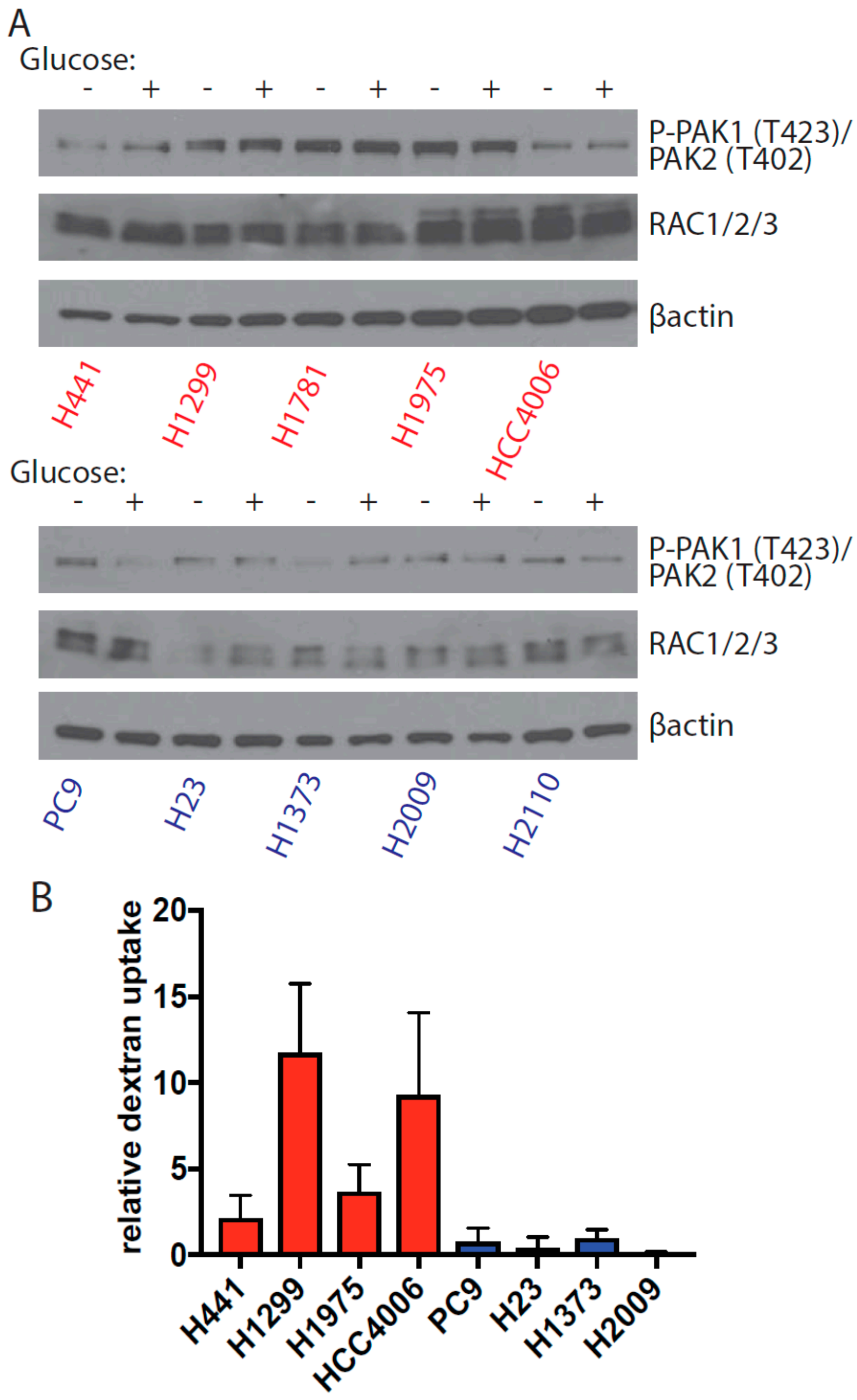

Figure S4. Rac signaling and macropinocytosis is not regulated by glucose. (**A**) Immunoblot analysis of Rac signaling activity via Pak autophosphorylation Thr-423 (Pak1)/Thr402 (Pak2). Glucose independent (red) and glucose addicted (blue) cells were cultured in the presence (+) or absence (−) of glucose for 6 h before lysate collection. (**B**) Cells cultured in full growth media were incubated with 1 mg/mL FITC-dextran for 1 h. Levels of FITC-dextran uptake in glucose independent (blue) and glucose addicted (red) cells was quantified and normalized to the cell line H1373.

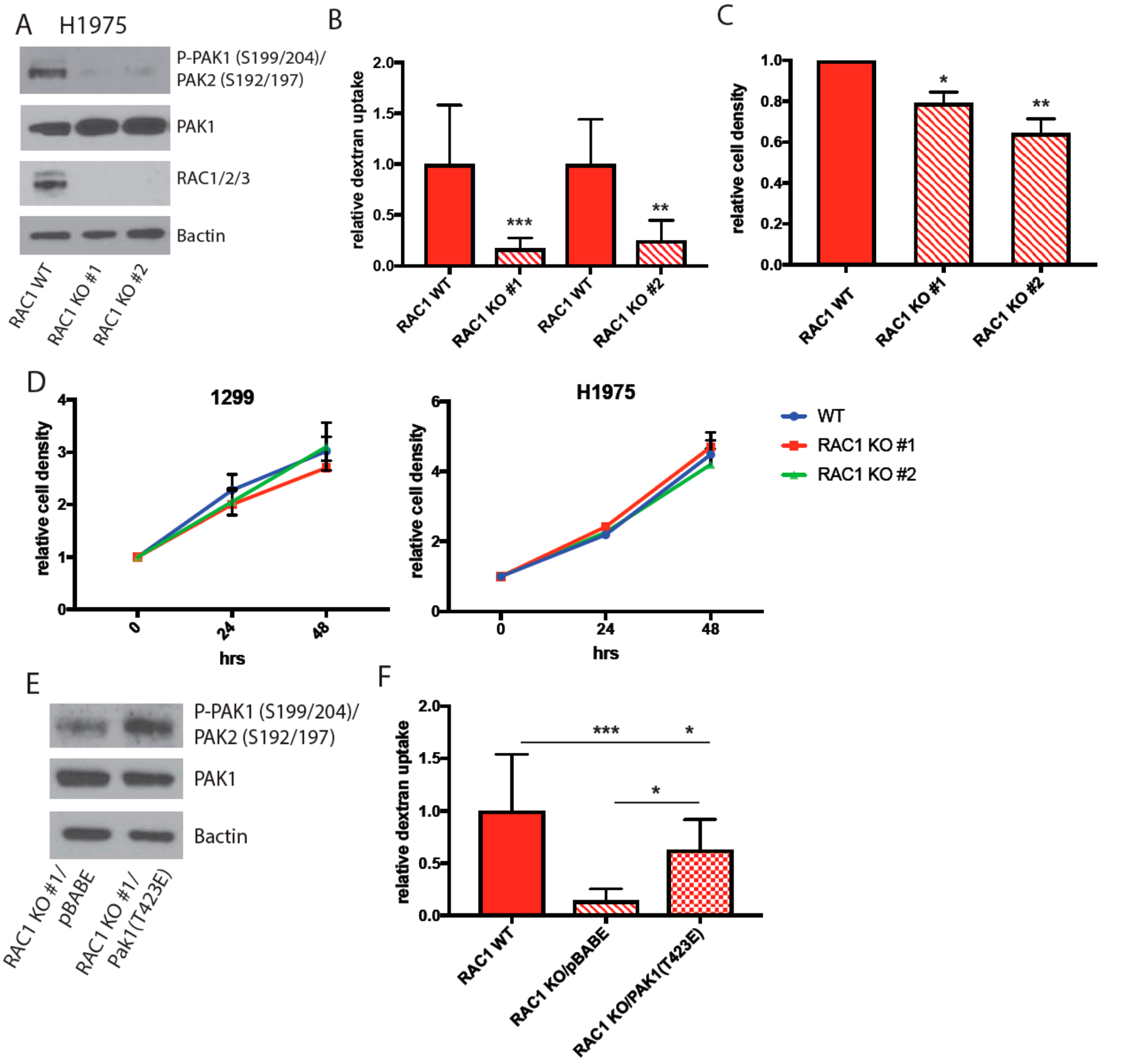

Figure S5. Rac1 regulates macropinocytosis in a PAK dependent manner to promote glucose independence. (**A**) Immunoblot analysis comparing Rac signaling in Rac1 wild-type (WT) and Rac1 knockout (KO) H1975 cells generated by CRISPR-Cas9. (**B**) Quantification of FITC-dextran uptake in glucose starved H1975 Rac1 WT and Rac1 KO cells. Data is normalized to Rac1 WT cells, error bars indicate +/- SD of 10 fields scored containing at least 10 cells. (**C**) Density of H1975 Rac1 WT and Rac1 KO cells cultured in GFM for 48 h. Data is normalized to Rac1 WT cells, error bars indicate +/- SEM of three independent experiments. For (**B**,**C**), significance was calculated using Student's *t*-test. (**D**) Density of Rac1 WT and Rac1 KO cells cultured in full growth media. Data is normalized to Rac1 WT cells, error bars indicate +/- SEM of at least two independent experiments. (**E**) Immunoblot analysis of Pak signaling in Rac1 KO. H1975 control cells or cells stably overexpressing the constitutive active Pak mutant Pak1 (T423E). (**F**) Quantification of FITC-dextran uptake in glucose starved Rac1 WT, Rac1 KO, and Rac1 KO/Pak1 (T423E) cells. Data is normalized to Rac1 WT cells; error bars indicate +/- SD of 10 fields scored containing at least 10 cells. Significance was calculated using ANOVA with Holm-Sidak multiple comparisons across cell lines. * $p < 0.05$, ** $p < 0.005$, *** $p < 0.0001$.

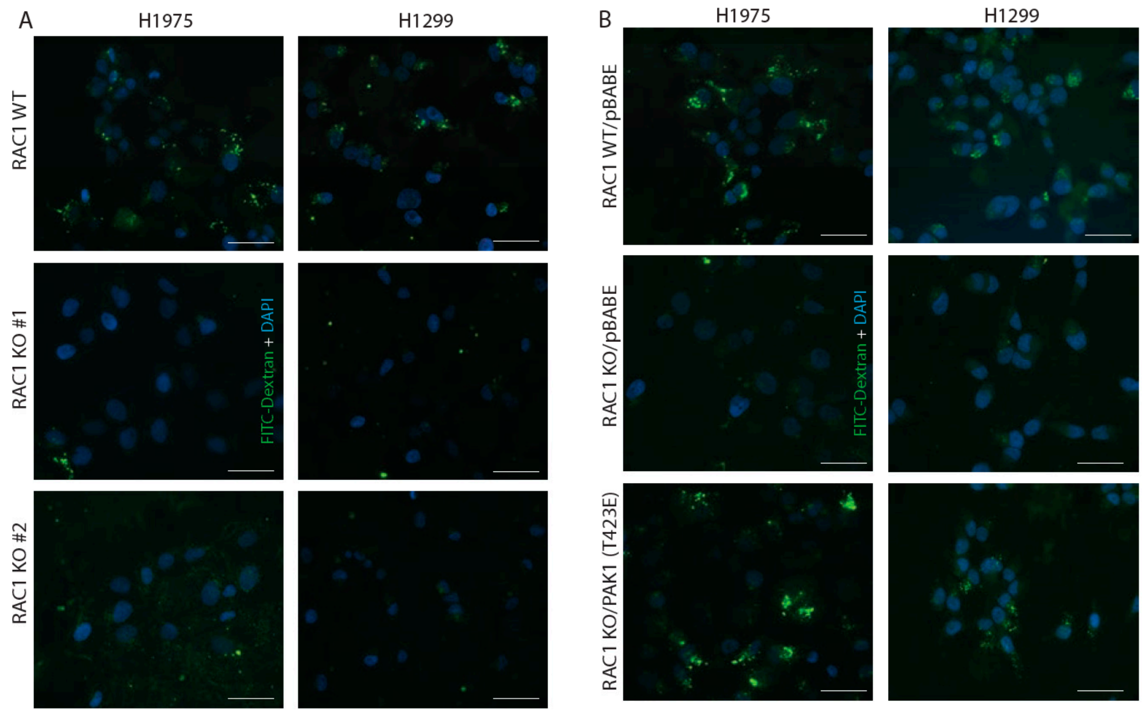

Figure S6. Rac1 activation of Pak regulates macropinocytosis. (**A**) Representative images of FITC-dextran internalization in Rac1 WT and Rac1 KO cells. Cells were cultured in GFM for 3 h prior to the addition of 1 mg/mL FITC-dextran for 1 h. Scale bar, 50 µm. (**B**) Representative images of FITC-dextran internalization in H1975 Rac1 WT, and Rac1 KO, and Rac1 KO/Pak1 (T423E) cells. Cells were cultured in GFM for 3 h prior to the addition of 1 mg/mL FITC-dextran for 1 h Scale bar, 50 µm.

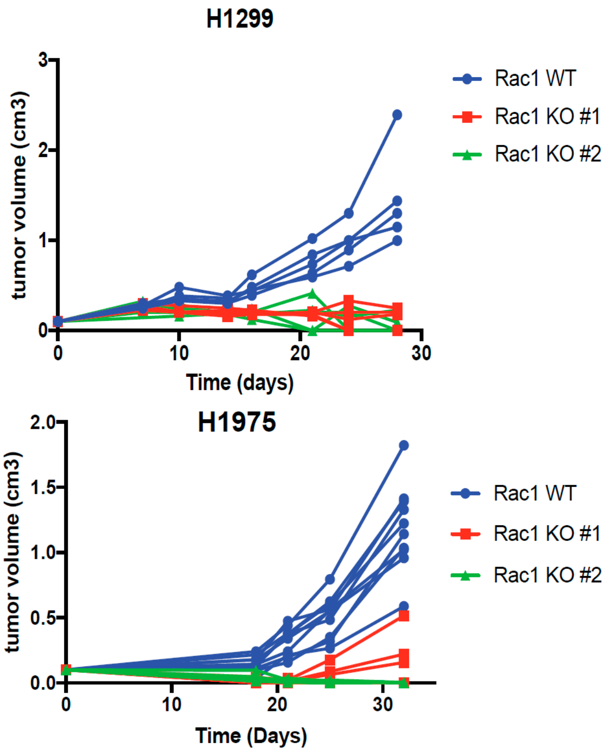

Figure S7. Rac1 is required for tumor formation of glucose independent cells. Rac1 WT and Rac1 KO H1299 and H1975 xenograft tumor volume was measured using calipers over time. H1299 xenografts: $n = 5$, H1975 xenografts: $n = 10$ except H1975 Rac1 KO #2: $n = 8$.

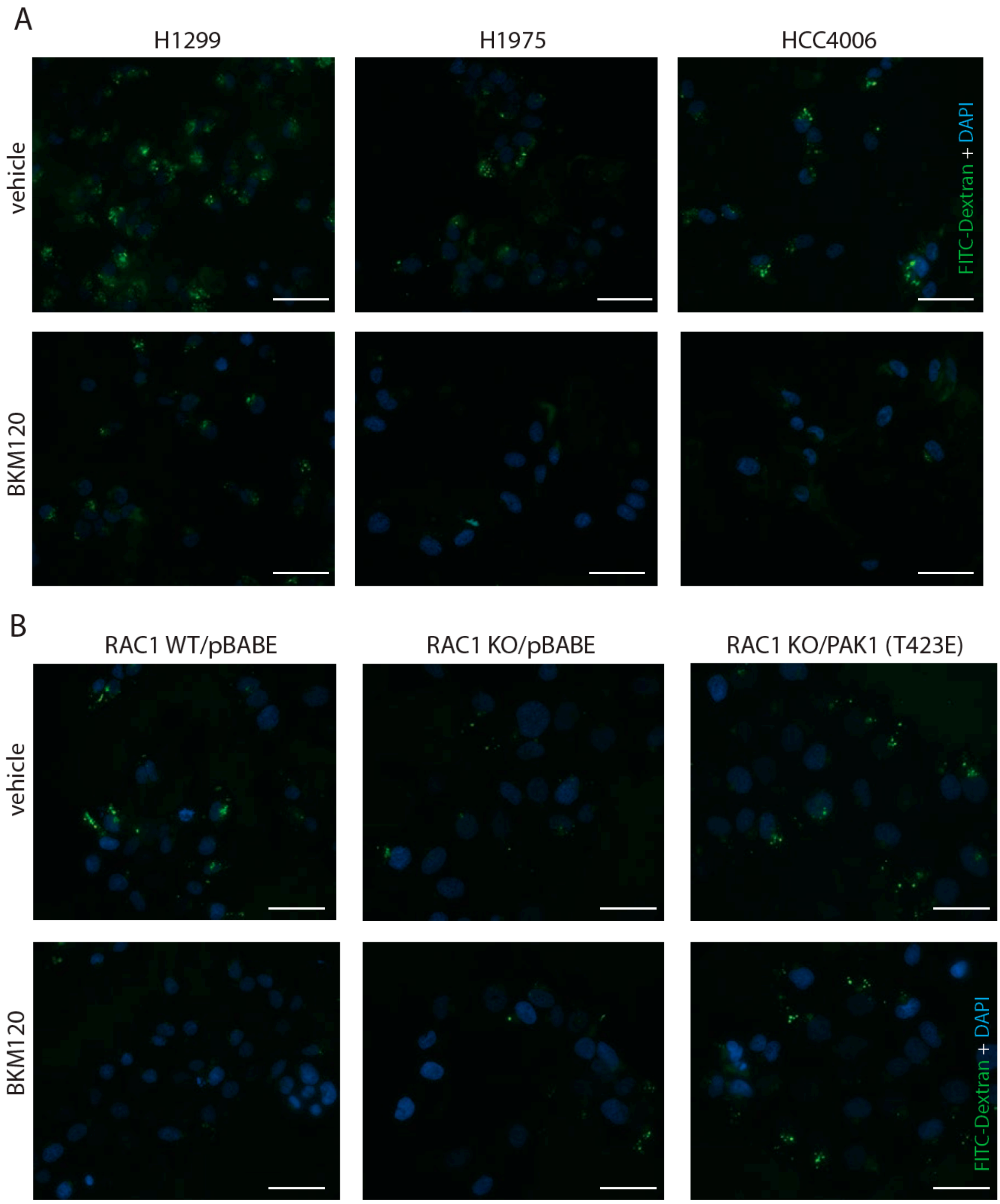

Figure S8. PI3K activates Rac-mediated macropinocytosis. (**A**) Representative images of FITC-dextran internalization in glucose starved cells treated with either vehicle or 1 μm BKM120 for 1 h prior to incubation with FITC-dextran. Scale bar, 50 μm. (**B**) Representative images of FITC-dextran internalization in H1975 Rac1 WT, Rac1 KO, and Rac1 KO/Pak1 (T423E) cells treated with either vehicle or 1 μm BKM120 in GFM for 1 h prior to incubation with FITC-dextran. Scale bar, 50 μm.

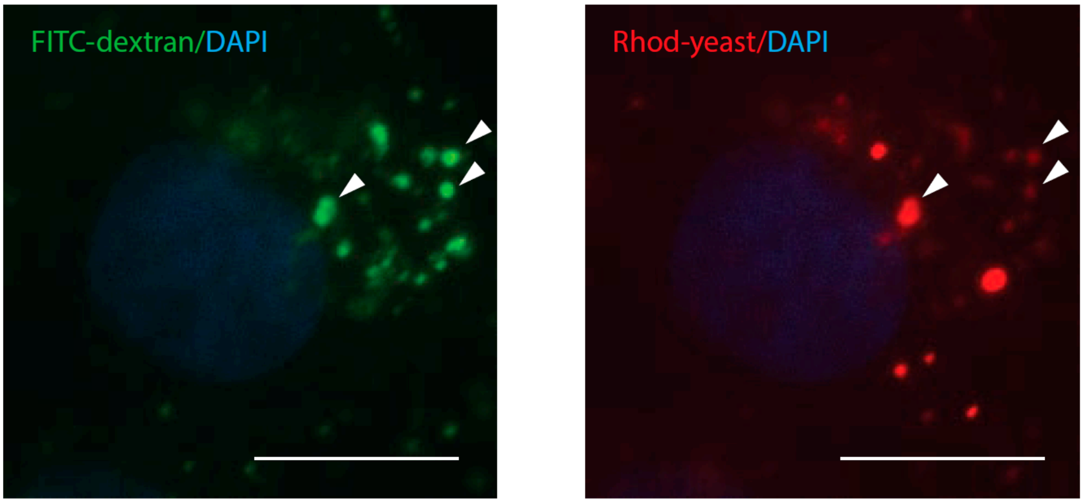

Figure S9. Co-localization of internalized Rhodamine-labeled yeast protein (red) with FITC-dextran (green) puncta in H1975 cells following 1 h incubation. Scale bar, 10 μm.

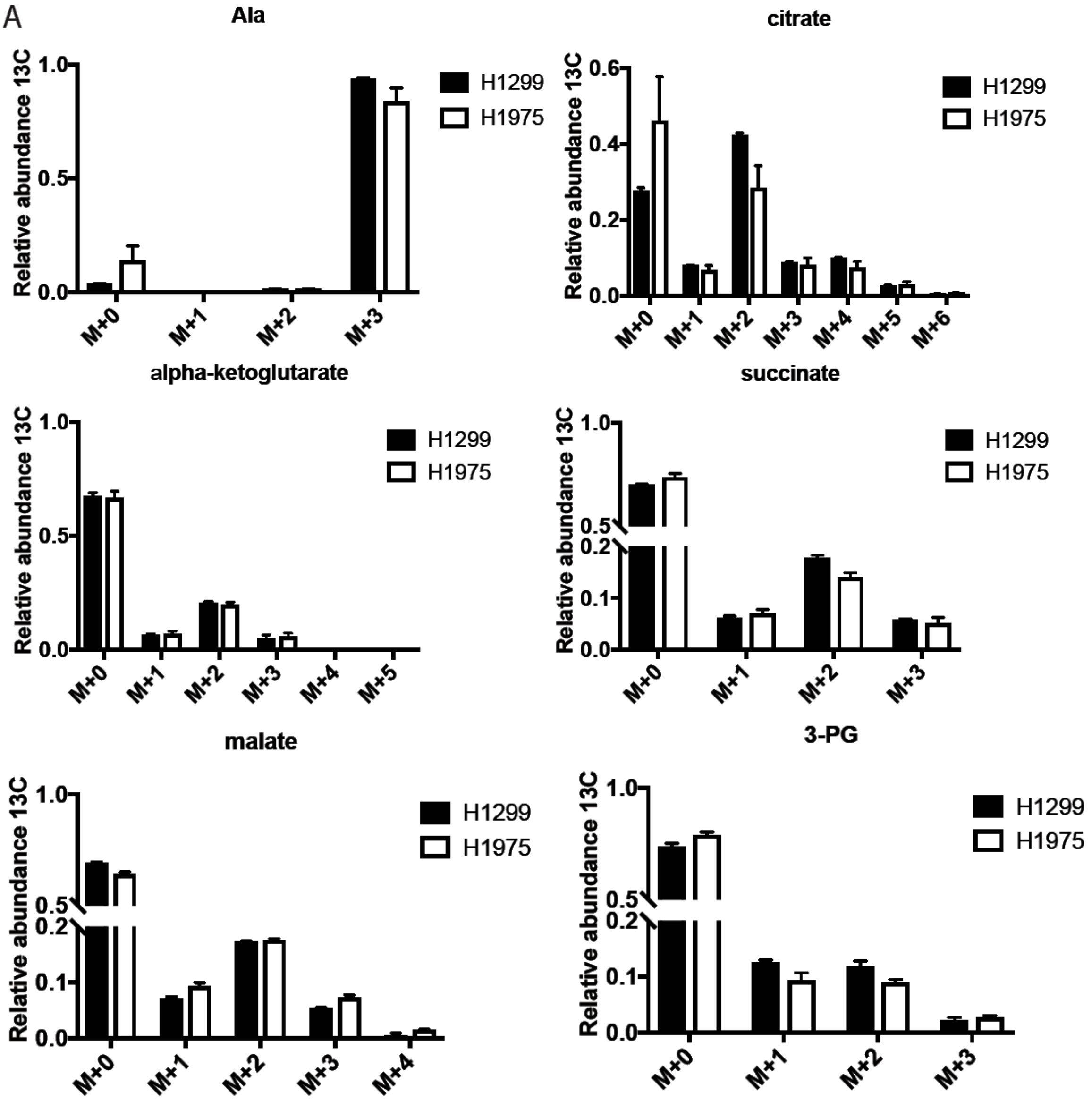

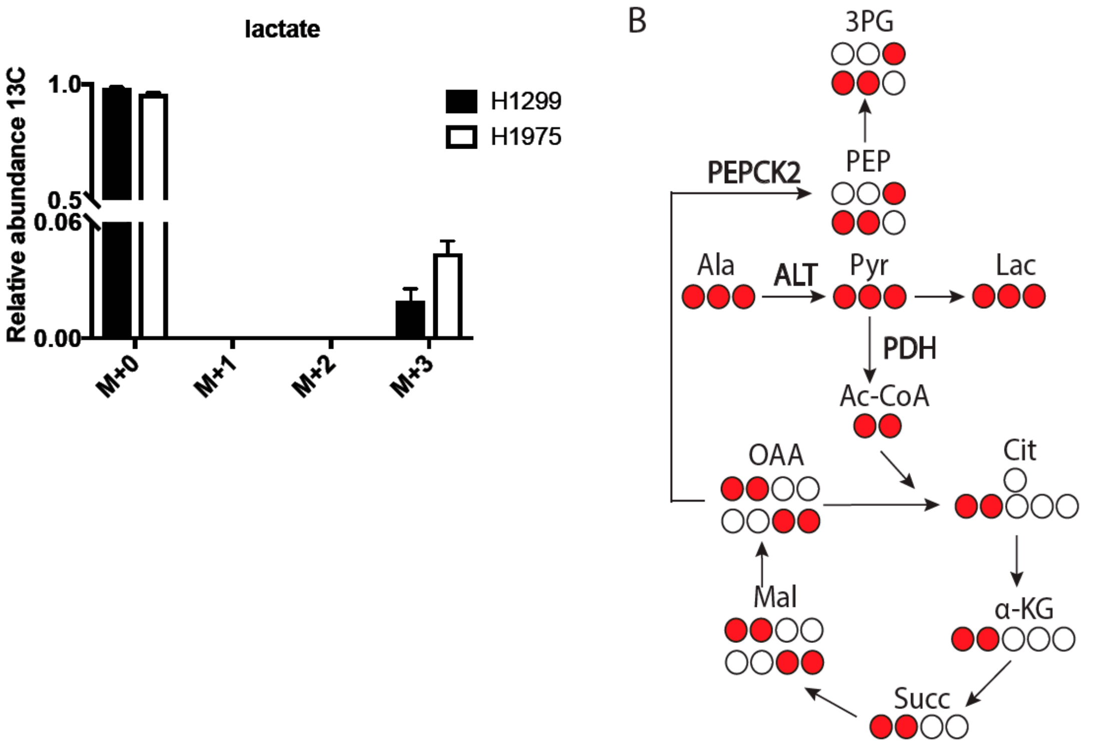

Figure S10. Alanine generates TCA cycle and gluconeogenic intermediates. (**A**) H1299 and H1975 cells were cultured in GFM supplemented with ^{13}C alanine flux for 24 h. Alanine flux was observed in TCA cycle and gluconeogenic metabolites as evident by incorporation of ^{13}C labeling. "M" refers to the number of labeled carbons detected, and values are presented as a ratio of ^{13}C labeled metabolite abundance to total metabolite abundance. Black bar = H1299, white bar = H1975. Error bars indicate +/- SD of three biological replicates. (**B**) Schematic of observed labeling patterns. Red circles represent 13C labeled carbons. Ala, alanine; Pyr, pyruvate; Lac, lactate; Ac-CoA, acetyl CoA; Cit, citrate; α-KG, α-ketoglutarate; Succ, succinate; Mal, malate; OAA, oxaloacetate; PEP, phosphoenolpyruvate; 3-PG, 3-phosphoglycerate.

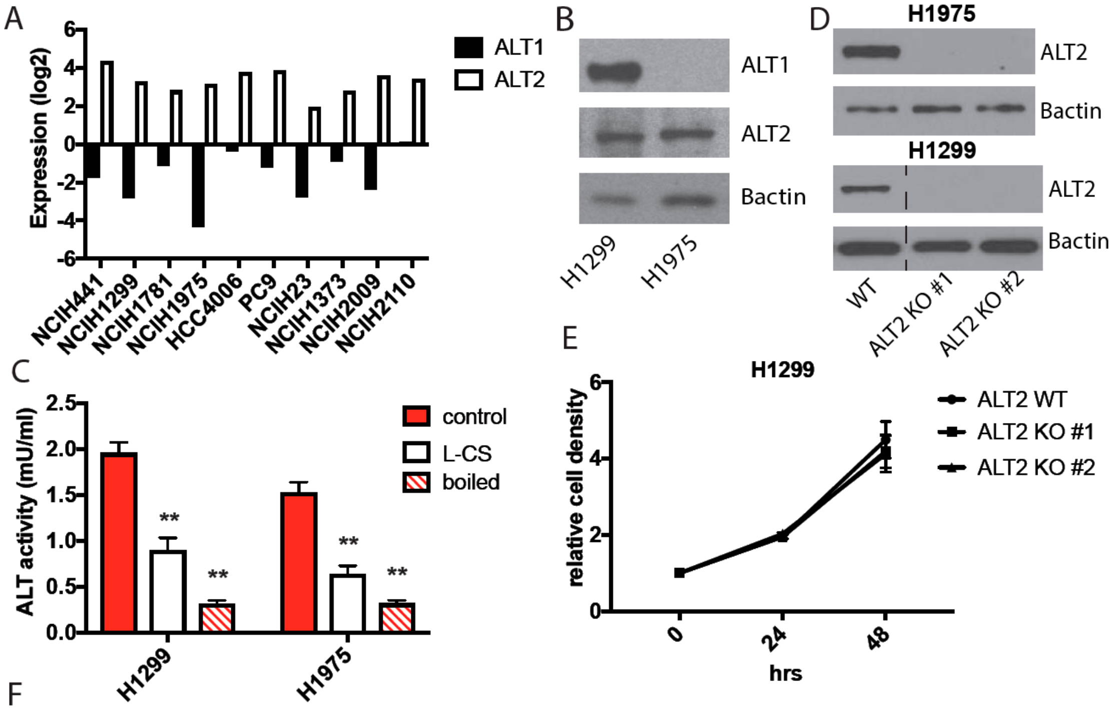

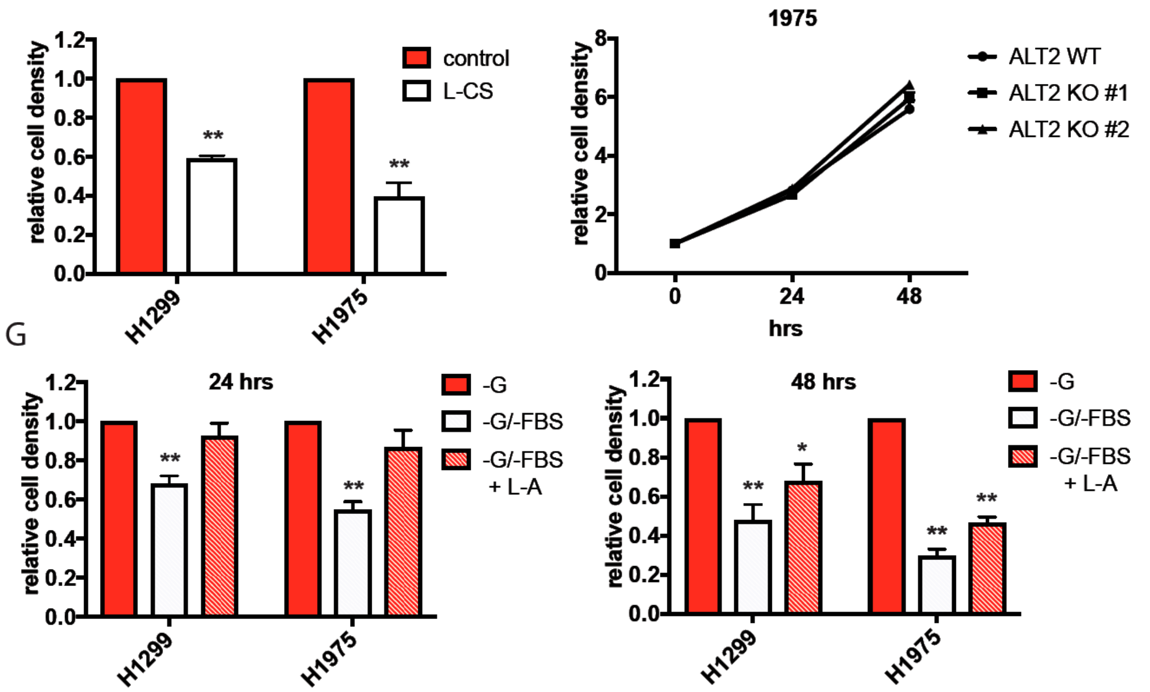

Figure S11. ALT2 is expressed in NSCLC cells and converts alanine to pyruvate to support glucose free survival. (**A**) Data from the Cancer Cell Line Encyclopedia was used to compare expression levels of ALT1 and ALT2 in lung cancer. (**B**) Immunoblot analysis of ALT1 and ALT2 levels in H1299 and H1975 cells. (**C**) ALT activity of H1299 and H1975 cell lysates that were treated with vehicle or 250 μm L-cyloserine (L-CS), or boiled. (**D**) Immunoblot analysis of ALT2 expression in ALT2 WT and ALT2 KO cells generated by CRISPR-Cas9 technology. (**E**) Density of ALT2 WT and ALT2 KO cells cultured in full growth media. Data is normalized to WT cells. (**F**) Relative density of glucose starved cells treated with vehicle or 250 μm L-CS for 48 h. Data is normalized to vehicle treated cells. For (**B**–**F**), significance was calculated using Student's *t*-test. (**G**) Relative density of glucose and serum starved cells treated with vehicle or 2 mm L-alanine (L-A) for 24 and 48 h. Data is normalized vehicle treated cells. Error bars: +/- SEM of three independent experiments. Significance was calculated using ANOVA with Holm-Sidak multiple comparisons to –G treated cells. * $p < 0.05$, ** $p < 0.005$.

© 2018 by the authors. Licensee MDPI, Basel, Switzerland. This article is an open access article distributed under the terms and conditions of the Creative Commons Attribution (CC BY) license (http://creativecommons.org/licenses/by/4.0/).
